# MeltingPlot, a user-friendly online tool for epidemiological investigation using High Resolution Melting data

**DOI:** 10.1186/s12859-021-04020-y

**Published:** 2021-02-18

**Authors:** Matteo Perini, Gherard Batisti Biffignandi, Domenico Di Carlo, Ajay Ratan Pasala, Aurora Piazza, Simona Panelli, Gian Vincenzo Zuccotti, Francesco Comandatore

**Affiliations:** 1 Department of Biomedical and Clinical Sciences “L. Sacco”, Pediatric Clinical Research Center “Romeo and Enrica Invernizzi”, Università Di Milano, 20157 Milan, Italy; 2 Department of Clinical, Surgical, Diagnostic and Pediatric Sciences, University of Pavia, Pavia, 27100 Italia; 3 Department of Pediatrics, Children’s Hospital Vittore Buzzi, Università Di Milano, Milan, Italy

**Keywords:** High Resolution Melting, Epidemiology, Bacterial typing, Real time surveillance, Nosocomial infection, Outbreak reconstruction, Web interface

## Abstract

**Background:**

The rapid identification of pathogen clones is pivotal for effective epidemiological control strategies in hospital settings. High Resolution Melting (HRM) is a molecular biology technique suitable for fast and inexpensive pathogen typing protocols. Unfortunately, the mathematical/informatics skills required to analyse HRM data for pathogen typing likely limit the application of this promising technique in hospital settings.

**Results:**

MeltingPlot is the first tool specifically designed for epidemiological investigations using HRM data, easing the application of HRM typing to large real-time surveillance and rapid outbreak reconstructions. MeltingPlot implements a graph-based algorithm designed to discriminate pathogen clones on the basis of HRM data, producing portable typing results. The tool also merges typing information with isolates and patients metadata to create graphical and tabular outputs useful in epidemiological investigations and it runs in a few seconds even with hundreds of isolates. Availability: https://skynet.unimi.it/index.php/tools/meltingplot/.

**Conclusions:**

The analysis and result interpretation of HRM typing protocols can be not trivial and this likely limited its application in hospital settings. MeltingPlot is a web tool designed to help the user to reconstruct epidemiological events by combining HRM-based clustering methods and the isolate/patient metadata. The tool can be used for the implementation of HRM based real time large scale surveillance programs in hospital settings.

## Background

The rapid typing of pathogens is pivotal to perform fast epidemiological investigations and to detect and block outbreaks. High-Resolution Melting (HRM) analysis is a single-step molecular biology technique able to determine the melting temperature of a PCR amplicon. The main output of an HRM assay is the melting curve, which indicates the denaturation level of the DNA amplicon in relation to temperature. The melting temperature is defined as the temperature in which half of the DNA duplex is dissociated. Considering that the melting temperature depends on the nucleotide composition of the amplicon, melting temperature can be used to discriminate sequence alleles. For each isolate, HRM analysis interrogates *n* specific genomic regions returning *n* melting temperatures, where each genomic region is defined by a specific PCR primer set. As stated above, The melting temperatures of each interrogated genomic region depend on its nucleotide composition. Consequently, melting temperatures can be used to cluster the isolates in a *n*-dimensional space. Previous works suggested that HRM data can be used for fast pathogen typing [1–4]. Recently, we developed a graph-based algorithm for isolate clustering on the basis of HRM temperatures and we showed that this approach is able to discriminate the most epidemiologically relevant clones of *Klebsiella pneumoniae* [4], one of the most important nosocomial pathogens world-wide [5]. In the same work, we compared this HRM *K. pneumoniae* typing protocol with Multi Locus Sequence Typing (MLST) and Whole Genome Sequencing (WGS) approaches: HRM typing protocol showed a discrimination power comparable to MLST on clinical *K. pneumoniae* isolates [4]. Additionally, in another work we showed that the protocol is highly reproducible and repeatable among instruments and operators [6].

Here we present MeltingPlot, a tool for rapid epidemiological investigation using HRM data. The tool implements an evolution of the clustering algorithm we already published [4]. Moreover, MeltingPlot merges HRM typing information with metadata of isolates and patients to get a comprehensive epidemiological investigation. MeltingPlot has a user-friendly web interface (the standalone command line version is also available) and it creates easy to read graphical and tabular outputs. The tool runs in a few seconds even with hundreds of isolates.

## Implementation

The flow of MeltingPlot can be divided in three main steps**:** HRM-based clustering/typing of isolates, prevalence analysis and transmission analysis. HRM-based clustering/typing is computed only on the basis of the High Resolution Melting (HRM) temperatures of the isolates amplicons, that are the only inputs needed for this step. Actually, the tool does not use any other information that can be derived from the melting curves, like the shape or the height of the curve. Indeed, in our experience, these features are more subjected to experimental noise than melting temperature so we are not using them to type the isolates. After the computation of the average HRM temperature of the technical replicates, the isolates are organized in a graph where the vertices represent the isolates and two vertices are connected if the difference of their average HRM temperatures is less or equal to 0.5 °C for each PCR primer set used in the HRM typing method. The graph is then decomposed into separate components (groups of connected vertices) and each one is then divided in clusters using the Edge Betweenness Clustering algorithm [7] implemented in the cluster_edge_betweenness function of the igraph R library [8]. Briefly, the betweenness centrality of each edge of the graph was computed as the number of shortest paths that go through the edge, and clusters were identified by gradually removing the edges with the highest betweenness centrality values. Therefore, high betweenness centrality values among two vertices indicates that the two vertices most probably do not belong to the same cluster and vice versa.

Furthermore, the betweenness centrality of a vertex was computed as the number of graph short paths passing that vertex. Hence, vertices with higher betweenness centrality values are those that connect two or more clusters. We used this parameter to identify vertices that were not strongly associated with a single cluster. Thus, vertices with normalized betweenness centrality values above a threshold were not assigned to any cluster and they were classified as “undetermined” by the tool (this threshold of normalized betweenness value can be set by the user, the default is 0.5).

Unfortunately, HRM-based clustering results obtained from different datasets are not directly comparable. To obtain comparable HRM typing results, the user can include in the analysis the HRM temperatures of a collection of reference strains: isolates previously analysed by the same HRM protocol and for which typing annotation is known (e.g. Sequence Type). When a reference collection is provided, MeltingPlot labels each cluster with the annotation of the reference isolates contained in it. For details see the Additional file 1.

Prevalence analysis and transmission analysis steps can be performed only when patients/isolates metadata is provided. In these steps the tool joins the HRM clustering results with the isolates’ metadata to create various outputs that depict the spreading of pathogen clones among wards and patients over time. For more details see the output files section below or the Additional file 1. MeltingPlot was developed in R and its dependencies are the libraries igraph [8], gplots [9], xlsx [10], ggplot2 [11], scales [12]. The user interface on the website was developed in PHP.

### Input file

Users are required to download and fill an xls template spreadsheet that contains four sheets: *HRM_temperatures*, *Isolates_metadata*, *Reference_isolates* and an *HELP_notes* sheet:

- *HRM_temperatures:* in this sheet the user has to report the high resolution melting (HRM) temperatures of the study isolates. This is the only mandatory data and it is used to perform the HRM-based clustering/typing analysis. If HRM experiments were performed using technical replicates, the users have to report all the replicate temperatures;
- *Isolates_metadata*: in this sheet the users can provide patients/isolates metadata, e.g. isolation date, isolation location (e.g. hospital ward) and an ID for the patients (e.g. Pz1, Pz2, …). This information is not mandatory for HRM isolates typing but it is required to perform the complete epidemiological investigation (i.e. prevalence analysis and transmission analysis);
- *Reference_isolates*: this sheet contains the HRM temperatures of the reference isolates and their annotation (e.g. Sequence Type). The reference isolates annotation will be used to label the clusters, making the obtained HRM typing results portable when the same reference collection is used.
- *HELP_notes*: this sheet contains important information about the rules for each column of the spreadsheet.

All the templates (the blank template, the templates with reference HRM temperature collections, and the example files) are available on the MeltingPlot webpage.

### Output files

MeltingPlot creates three groups of plot files (in PDF and PNG format), one for each step of the analysis: HRM-based clustering/typing, prevalence analysis and transmission analysis. The HRM-based clustering/typing plot group includes the isolates graph (where each isolate is colored on the basis of its cluster) and a heatmap showing the HRM temperatures and the isolates clusters. In the isolates graph each vertex is an isolate and two isolates are connected as described above. The last two groups are created when metadata of the isolates is provided. The prevalence analysis plot group includes bar plots showing the distribution of the clusters over time in the different locations. The transmission analysis plot group contains a patient timeline and a patient-to-patient graph. In the latter, two patients are connected when two isolates belonging to the same HRM cluster were collected from both patients. The edge is thicker when the isolates were collected in the same location (e.g. ward) within a number of days set by the user (7 by default). Thus, thicker edges highlight most probable transmission events. MeltingPlot also produces xls spreadsheets containing the isolates HRM clusters and metadata. See Additional file 1 for details.

## Results

**Fig. 1:**
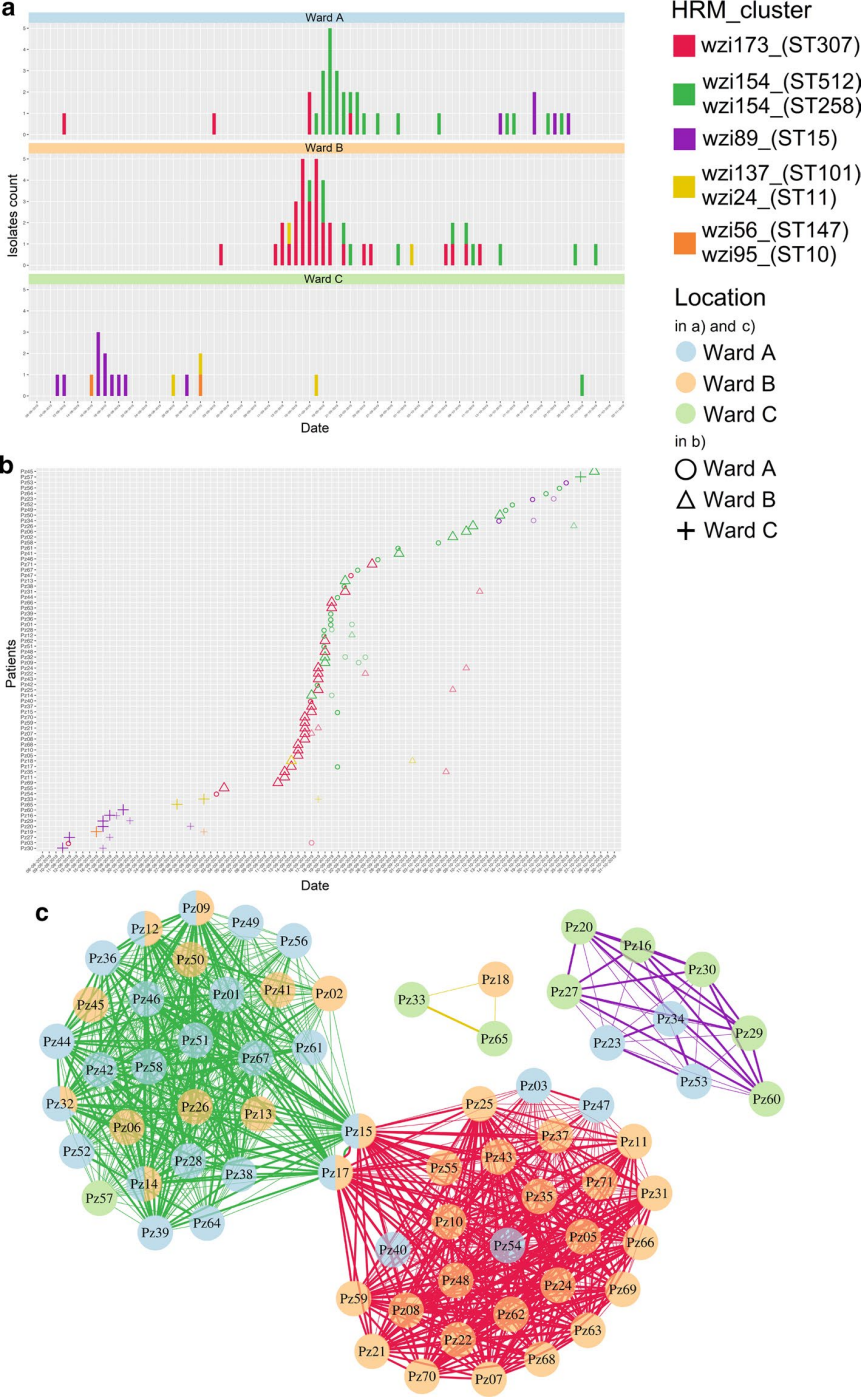
MeltingPlot Output, example of an epidemiological investigation. Here we report the three most significant MeltingPlot output plots obtained on the simulated dataset. The plots were selected to show the power of the HRM-based epidemiological investigation performed by the tool. Higher resolution images are available in the Additional file 1. **a** Prevalence analysis: the plot shows the number of isolates collected from each hospital ward over time. Each HRM cluster is represented with a different color. This analysis allows the detection of the pathogen clones emergence in the hospital setting. **b** Patients’ timeline: each row refers to a patient and the symbols represent isolates. The shape of the symbols report the location where the isolates were collected while the colors indicate the HRM cluster. **c** Patient-to-patient graph: each vertex represents a patient and two vertices are connected if isolates belonging to the same HRM cluster were collected from both patients. Vertices are reported as pie charts and colors show the locations (wards) where the isolates of the patients were collected. The edges of the graph are thicker if the isolates from the same HRM cluster were collected within seven days (this threshold can be defined by the user) from the same location. This plot can help to identify the transmission routes of the pathogen in the hospital setting

To show the capability of MeltingPlot, we simulated a complex large *Klebsiella pneumoniae* nosocomial outbreak (100 outbreak isolates) sustained by multiple clones spread in different wards, a situation comparable to real large nosocomial outbreaks [13]. The isolates were simulated using HRM temperatures retrieved from a dataset of *K. pneumoniae* isolates previously analyzed in our laboratory. We simulated an outbreak scenario sustained by five different clones: three major clones that caused the outbreak and two sporadic clones. Thus we included in the dataset the temperature of previously HRM typed isolates belonging to five highly epidemiologically relevant clusters: *wzi173_(ST307)*, *wzi154_(ST512/ST258)*, *wzi89_(ST15), wzi137_(ST101)/wzi24_(ST11)* and *wzi56_(ST147)/wzi95_(ST10)*; the first three causing the outbreaks and the last two being sporadic. We also simulated the metadata of the isolates to obtain a complex outbreak with three wards showing different epidemiological scenarios (see Fig. 1 for more detail). We run MeltingPlot using the 100 outbreak isolates dataset and a collection of 18 reference isolates previously typed by HRM and WGS [4] (Reference_isolates sheet in the input file, see above; the Reference_isolates temperature collection is also available on the tool web site). In our outbreak simulation, the HRM typing of the 100 isolates would be performed after every pathogen isolation during the entire outbreak period (~ 3 months). The entire epidemiological investigation using this HRM typing protocol would cost ~ 500 euros while it would cost ~ 5000€ using Multi Locus Sequence Typing (MLST) and ~ 10,000€ using Whole Genome Sequencing (WGS). As expected, Melting Plot results showed that the outbreak is sustained by three major isolate clusters (Fig. 1). MeltingPlot labelled these clusters as *wzi173_(ST307)* (in red), *wzi154_(ST512/ST258)* (in green) and *wzi89_(ST15)* (in violet) using the annotation given by the user for the reference isolates. Most of the infections are caused by two pathogen clusters (green and red), each one associated with a single ward: the green one with Ward A and the red one with Ward B. A smaller cluster (in violet) caused an outbreak in Ward C at the beginning of the investigated period. The patient’s timeline and the patient-to-patient graph clearly show that two patients (Pz15 and Pz17) were infected by isolates of both the red and green clusters and they also crossed the wards A and B: this highlights two possible pathogen transmission routes among the wards. A complete description of each output file is available in the Additional file 1. MeltingPlot is available online at https://skynet.unimi.it/index.php/tools/meltingplot/. The source code for the stand alone version is available at https://github.com/MatteoPS/MeltingPlot.

## Discussion

High Resolution Melting (HRM) is a fast and inexpensive molecular biology technique [2] applicable to pathogen typing and suitable for large scale surveillance programmes as well as for fast outbreak reconstruction [1, 3]. In this work we propose MeltingPlot, a tool that allows to perform epidemiological investigation and transmission analysis using HRM data.

The tool implements an algorithm for the HRM-based clustering that groups isolates on the basis of their melting temperatures. Unfortunately, the HRM-based clusters obtained from different collections of isolates are not directly comparable. To overcome this limitation we made MeltingPlot able to include in the clustering analysis the melting temperatures of a collection of reference isolates. MeltingPlot uses the reference isolates as a guide to label the obtained isolates clusters. On this way, MeltingPlot results obtained from different isolates collections become comparable.

Furthermore, MeltingPlot performs complete epidemiological investigations merging HRM clustering results with isolates/patients metadata. It produces easy-to-read graphical representations and tabular files (see Results section) useful to reconstruct epidemiological scenarios and to identify pathogen transmission routes.

The HRM typing is cost saving and it can be carried out using instruments usually present in hospital microbiology laboratories and by not highly specialized personnel. MeltingPlot also eases the HRM data analysis for epidemiological investigation. In our opinion, the implementation of HRM typing can improve nosocomial surveillance programs with a limited impact on the hospital policies in terms of costs, workload and personnel involved.

MeltingPlot is available in stand-alone and web versions. The web interface makes the tool user-friendly and the user has only to upload the data into an xls template spreadsheet. MeltingPlot analyses hundreds of isolates in a few seconds.

## Conclusions

HRM technique allows pathogen typing in a few hours and ~ 5 euros per sample. Despite this, the mathematical/informatics skills required for the analysis and interpretation of HRM results limit the application of HRM typing protocols in hospital real time surveillance. MeltingPlot is a user-friendly tool that facilitates the application of HRM to real time large scale surveillance programs in hospital settings.

## Availability and requirements

**Project name**: MeltingPlot.

**Project home page**: https://skynet.unimi.it/index.php/tools/meltingplot/

**Operating system(s)**: Platform independent.

**Programming language**: R, PHP.

**Other requirements**: Any web browser.

**License:** GPL.

**Any restrictions to use by non-academics:** none.

## Supplementary Information

**Additional file 1:** This file contains a detailed description of the clustering algorithm, of the input file and of each output file created by the tool.

## Data Availability

The tool described here, the template input file and the example files are freely available at https://skynet.unimi.it/index.php/tools/meltingplot/. The source code is available in the GitHub repository https://github.com/MatteoPS/MeltingPlot.

## Abbreviations

HRM: High Resolution Melting
PCR: Polymerase Chain Reaction
MLST: Multi-Locus Sequence Typing
WGS: Whole Genome Sequencing
