# Supplementary material for: MeltingPlot, a user-friendly online tool for epidemiological investigation using High Resolution Melting data
Source: BMC Bioinformatics. 2021 Feb 18;22:76. doi: 10.1186/s12859-021-04020-y (PMC7891011; doi:10.1186/s12859-021-04020-y)
Supplement: Supplementary file 1 — Additional file 1: This file contains a detailed description of the clustering algorithm, of the input file and of each output file created by the tool. [file 12859_2021_4020_MOESM1_ESM.pdf]

MeltingPlot, a user-friendly online tool for epidemiological investigation using High Resolution Melting data

Matteo Perini[1], Gherard Batisti Biffignandi[2], Domenico Di Carlo[1], Ajay Ratan Pasala[1], Aurora Piazza[2], Simona Panelli[1], Gian Vincenzo Zuccotti[1,3], and Francesco Comandatore[1,*]

[1]Department of Biomedical and Clinical Sciences “L. Sacco”, University of Milan, Pediatric Clinical Research Center “Romeo and Enrica Invernizzi”, Milan, 20157, Italy; [2]Department of Clinical, Surgical, Diagnostic and Pediatric Sciences, University of Pavia, Pavia, 27100, Italia; [3]Department of Pediatrics, Children's Hospital Vittore Buzzi, University of Milan, Italy;

*Corresponding author. email: francesco.comandatore@unimi.it

SUPPLEMENTARY MATERIAL

Clustering algorithm **2**

Input file **3**

Output files **5**

HRM-based clustering/typing 6

1a) Isolates graph 6

1b) Isolates heatmap 7

Prevalence analysis 8

2a) Isolates location (daily) 8

2b) Isolates location (monthly) 9

2c) Positivizations location (daily) 10

2d) Positivizations location (monthly) 11

2e) Positivizations global (daily) 12

2f) Positivizations global (monthly) 13

Transmission analysis 14

3a) Patients timeline 14

3b) Patient-to-patient graph 15

Tabular outputs 16

Isolates cluster table 16

Isolates cluster and patients table 17

References **18**

Clustering algorithm

The isolates are clustered by a graph-based method. First, the average melting temperature (aTm) is computed as the arithmetic mean of the technical replicates. Each isolate will be represented by *n* aTms, where *n* is the number of PCR primer sets used in the HRM typing protocol. Then, the isolate graph is built connecting two isolates when each of the *n* aTms of the two isolates do not range above 0.5°C (aTm difference <= 0.5°C, a threshold established in Perini et al. 2020 [1]). Subsequently, the graph is decomposed in separate components and for each component the community structure is detected by the Girvan–Newman algorithm [2] implemented in the "cluster_edge_betweenness" function (https://igraph.org/r/doc/cluster_edge_betweenness.html) of the R library igraph [3]. The algorithm measures the centrality of the vertices in the graph: a vertex that connects two distinct clusters will have a higher betweenness value. The "cluster_edge_betweenness" function performs a hierarchical clustering based on these values, clustering together the communities of vertices connected with low betweenness values. These communities will represent the HRM-based isolate clusters. Furthermore, a betweenness value is assigned to each vertex and a edge betweenness value is assigned to each edge by the "betweenness" and the "edge_betweenness" functions of igraph (https://igraph.org/r/doc/betweenness.html). These values are represented in the isolate graph as vertex size (smaller vertex = higher betweenness value) and edge thickness (thinner edge = higher edge betweenness value). Moreover, when a vertex has a normalized betweenness value above 0.5 it is not assigned to any cluster and it is classified as "undetermined" by the tool. The betweenness values are also reported in the tabular outputs. The normalized betweenness threshold can be set by the user.

Input file

ID_isolate	Primers_set	T1	T2	T3
Is01	wzi-3	84	84	84
Is01	wzi-4	84	84	84
Is10	wzi-3	84	84	84
Is10	wzi-4	82.5	82.5	82.5
Is05	wzi-3	83	82	82
Is05	wzi-4	84	85	85
Is11	wzi-3	84	84	84
Is11	wzi-4	82.5	82.5	82.5
Is12	wzi-3	84	84	84
Is12	wzi-4	84	84	84
Is13	wzi-3	84	84	84
Is13	wzi-4	84	84	84
Is14	wzi-3	84	84	84
Is14	wzi-4	84	84	84
Is15	wzi-3	84	84	84
Is15	wzi-4	82.5	82.5	82.5
Is16	wzi-3	84	84	84
Is16	wzi-4	82.5	82.5	82.5
Is17	wzi-3	84	84	84
Is17	wzi-4	84	84	84
Is19	wzi-3	84	84	84
Is19	wzi-4	84	84	84
Is02	wzi-3	84	84	84
Is02	wzi-4	84	84	84
Is20	wzi-3	84	84	84
Is20	wzi-4	82.5	82.5	82.5
[illegible]	[illegible]	[illegible]	[illegible]	[illegible]

HRM_temperatures | Isolates_metadata | Background_strains | HELP_notes

The input file is a xls spreadsheet.

The template is available here:
https://skynet.unimi.it/wp-content/uploads/MeltingPlot/MeltingPlot_Template.xls.

The example file used in the work is available here:
https://skynet.unimi.it/wp-content/uploads/MeltingPlot/files_supplementary_material/big_example.xls

Other Example files and templates with reference isolates temperatures from different instrument models are available at the tool's website: https://skynet.unimi.it/index.php/tools/meltingplot/

Each xls input file contains 4 sheets: "*HRM_temperatures*", "*Isolates_metatada*", "*Reference_isolates*", "*HELP_notes*".

- ***HRM_temperatures***
  The user needs to add here the temperatures measured by the PCR-HRM experiments performed on the isolates. Each row contains the temperatures relative

to one isolate and one primer set. (when multiple primer sets are used for the PCR-HRM experiment, multiple rows per isolate should be used).

The fields are:

- *ID_isolate*: unique ID for each isolate.
- *Primer_set:* name of the primer set used for the single experiment.
- *T1,T2,T3...*: measured melting temperatures. The temperatures from each technical replicate (T1, T2, T3, ...) of the single experiment should be reported. If a different number of replicates were performed, the user can add or remove columns. The number of replicates must always be specified before launching the analysis.

- ***Isolates_metatada***

  In this sheet the user can specify from which patient each isolate was collected.

  The fields are:

  - *ID_isolate*: unique ID for each isolate. (must be the exactly the same ID reported in the *HRM_temperature* sheet).
  - *ID_patient:* unique ID for each patient.
  - *Date:* isolate collection date
  - *Location:* location of the isolate collection (e.g ward)

- ***Reference_isolates***

  In the sheet the user can report the temperature and the information about the reference strains. The information provided here will be used to name the clusters.

  The fields are:

  - *ID_isolate*: unique ID for each reference isolate.
  - *Annotation:* characteristic feature of the reference isolate (e.g. Sequence type). This information will be used to name the cluster during the HRM-based clustering/typing of the isolates
  - *T1,T2,T3...*: measured melting temperatures. The temperatures from each technical replicate of the single experiment should be reported. If a different number of replicates were performed, the user can add or remove columns. The number of replicates must always be specified before launching the analysis.

- ***HELP_notes***

  This sheet contains a summary of the rules and information necessary to fill the file.

Output files

Click on the file name to read the output descriptions (or see below). All the output images refer to the example dataset used in the work. The example dataset file is available here: https://skynet.unimi.it/wp-content/uploads/MeltingPlot/files_supplementary_material/big_example.xls

The graphical outputs are:

1. HRM-based clustering/typing

1a) Isolates graph
1b) Isolates heatmap

2. Prevalence analysis

2a) Isolates location (daily)
2b) Isolates location (monthly)
2c) Positivization location (daily)
2d) Positivization location (monthly)
2e) Positivization global (daily)
2f) Positivization global (monthly)

3. Transmission analysis

3a) Patients timeline
3b) Patients graph

The tabular outputs are:

Isolates cluster table
Isolates cluster and patients table

1. HRM-based clustering/typing

1a) Isolates graph

Each vertex represents an isolate. The isolates are clustered on the basis of the edge betweenness as described in the clustering algorithm section of this document. When the reference isolates are provided, the clusters take the name of the reference isolates that they may contain. Vertices sizes depend on the betweenness values and edges thickness depends on the edge betweenness values. As a result, smaller vertices indicate uncertain clustering of the isolates and thinner edges connect isolates that unlikely belong to the same cluster.

High resolution file:
https://skynet.unimi.it/wp-content/uploads/MeltingPlot/files_supplementary_material/pdf_1a_big_isolates_graph.pdf

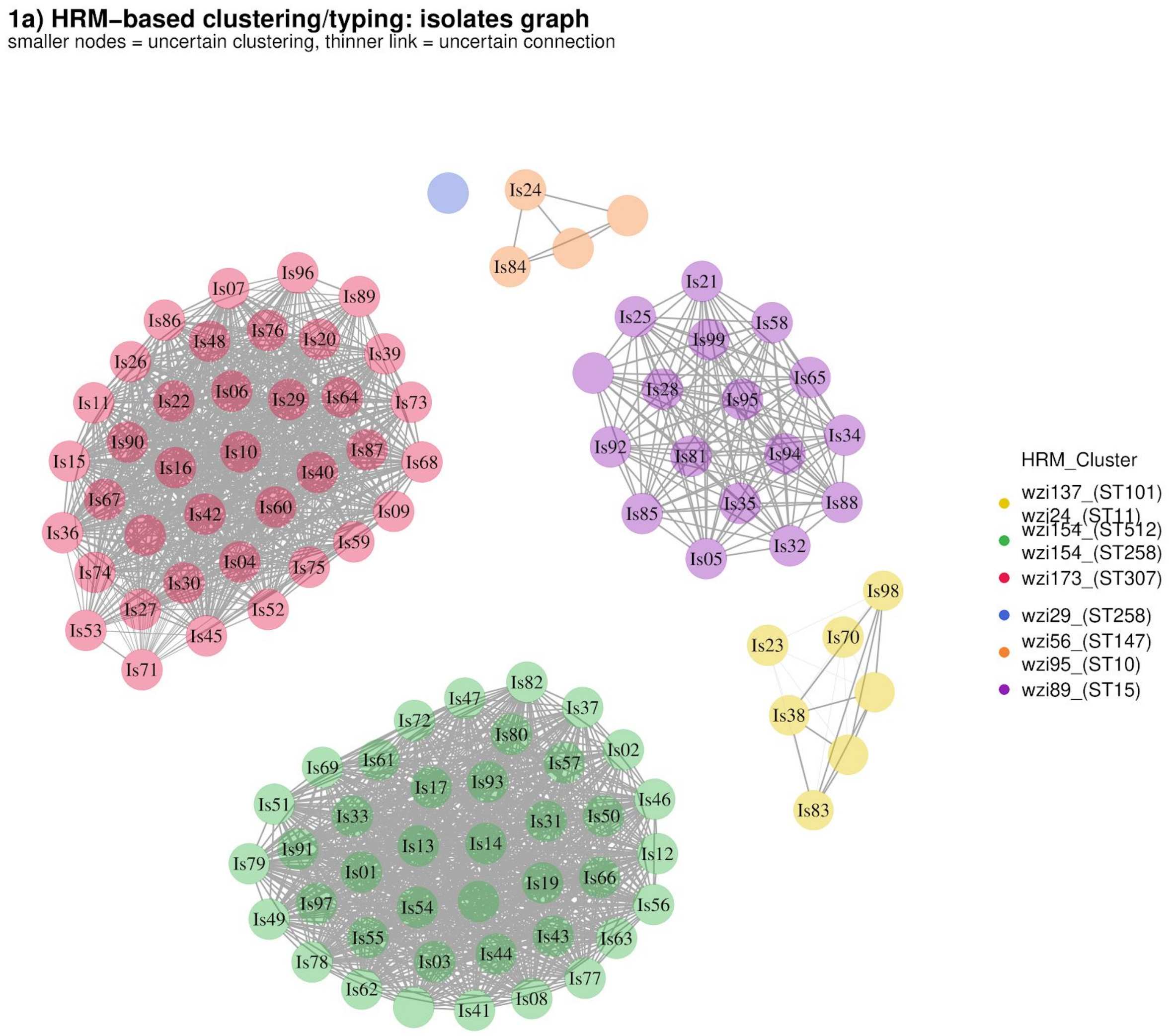

1b) Isolates heatmap

The heatmap shows all the melting temperatures provided and groups the isolates by clusters. The reference strains’ names are gray to easily distinguish them from the other isolates.

High resolution complete file:
https://skynet.unimi.it/wp-content/uploads/MeltingPlot/files_supplementary_material/pdf_1b_big_isolates_heatmap.pdf

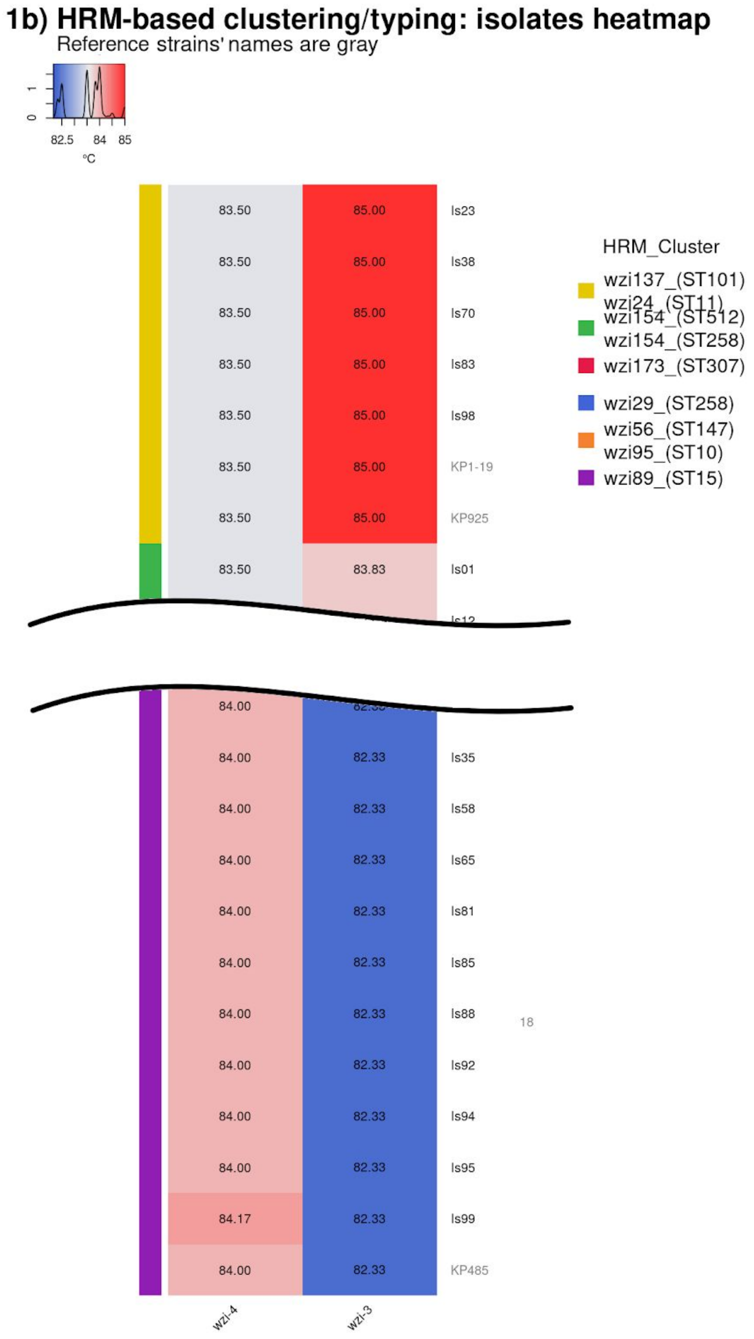

2. Prevalence analysis

2a) Isolates location (daily)

The bar charts show the location and the collection day for each isolate. The position on the x axis correspond s to the isolation date. The y axis is divided in panels that correspond to the locations (wards) provided, a color is assigned to each location and it is shown in the panel's title band.

High resolution file:
https://skynet.unimi.it/wp-content/uploads/MeltingPlot/files_supplementary_material/pdf_2a_big_isolates_location_daily.pdf

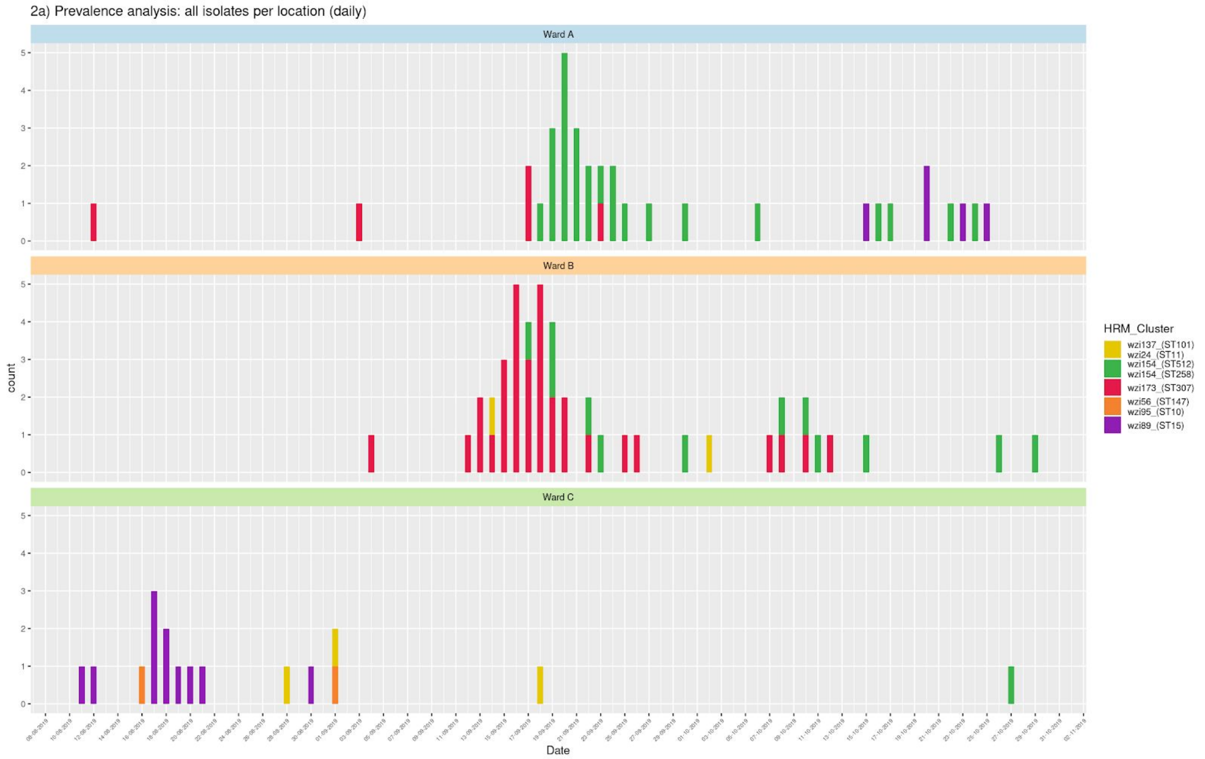

2b) Isolates location (monthly)

The bar charts show the location and the collection month for each isolate. This plot is created only if the collection date range is above 45 days. The position on the x axis corresponds to the isolation month. The y axis is divided in panels that correspond to the locations (wards) provided, a color is assigned to each location and it is shown in the panel's title band.

High resolution file:
https://skynet.unimi.it/wp-content/uploads/MeltingPlot/files_supplementary_material/pdf_2b_big_isolates_location_monthly.pdf

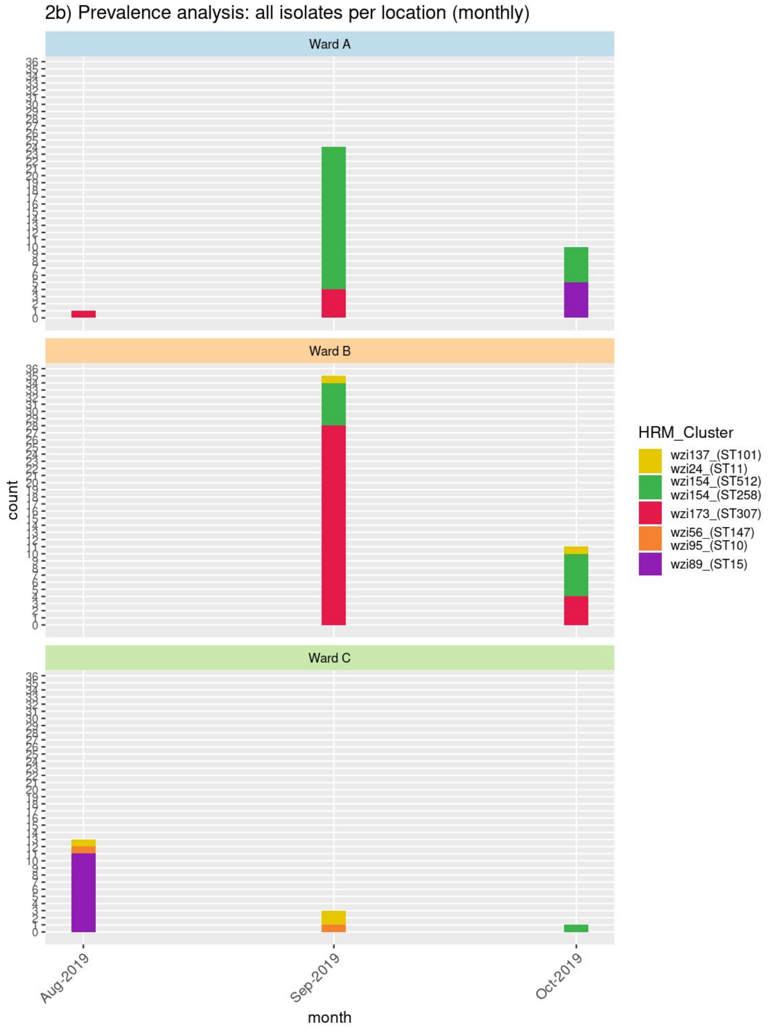

2c) Positivizations location (daily)

The bar charts show the location and the collection day for each positivization. A positivization corresponds to the first time a patient is found positive to an isolate of a cluster. When two isolates of different clusters are isolated from a patient, that patient will have two positivization events (one per cluster). The position on the x axis corresponds to the positivization day. The y axis is divided in panels that correspond to the locations (wards) provided, a color is assigned to each location and its shown in the panel's title band.

High resolution file:
https://skynet.unimi.it/wp-content/uploads/MeltingPlot/files_supplementary_material/pdf_2c_big_positivization_location_daily.pdf

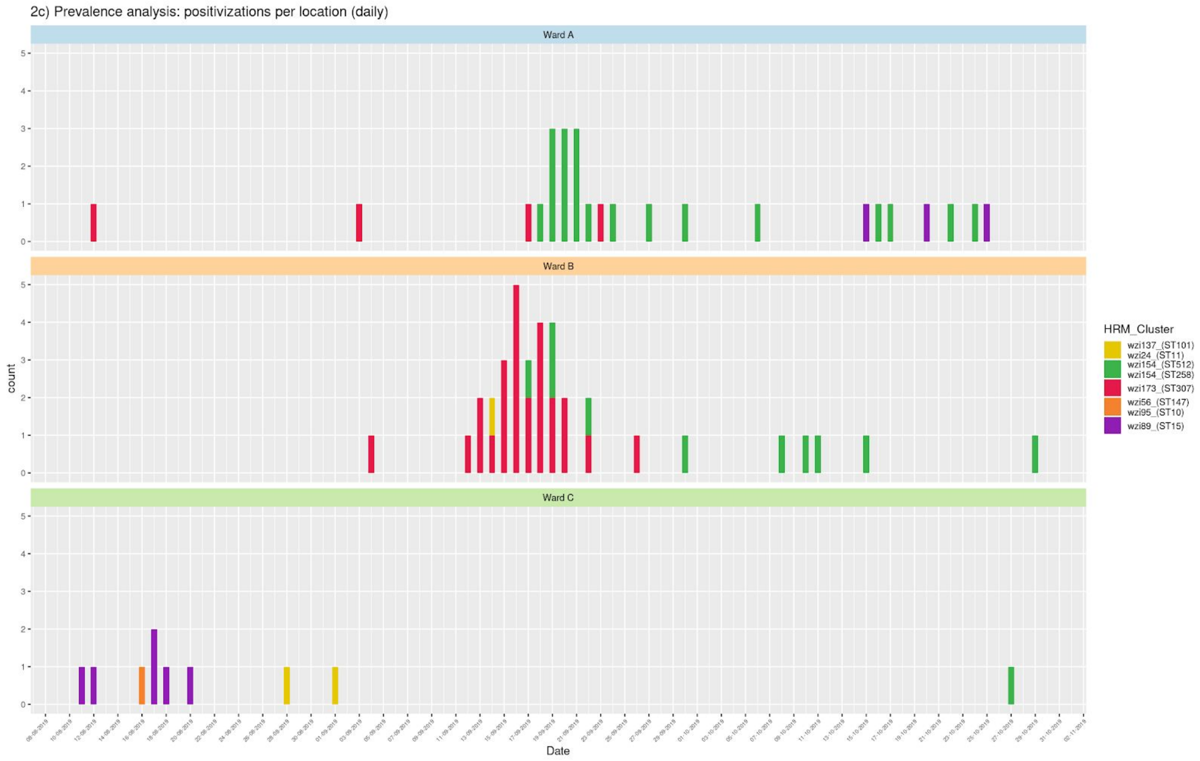

2d) Positivizations location (monthly)

The bar charts show the location and the collection month for each positivization. This plot is created only if the collection date range is above 45 days. A positivization corresponds to the first time a patient is found positive to an isolate of a cluster. When two isolates of different clusters are isolated from a patient, that patient will have two positivization events (one per cluster). The position on the x axis corresponds to the positivization month. The y axis is divided in panels that correspond to the locations (wards) provided, a color is assigned to each location and it is shown in the panel's title band.

High resolution file:
https://skynet.unimi.it/wp-content/uploads/MeltingPlot/files_supplementary_material/pdf_2d_big_positivization_location_monthly.pdf

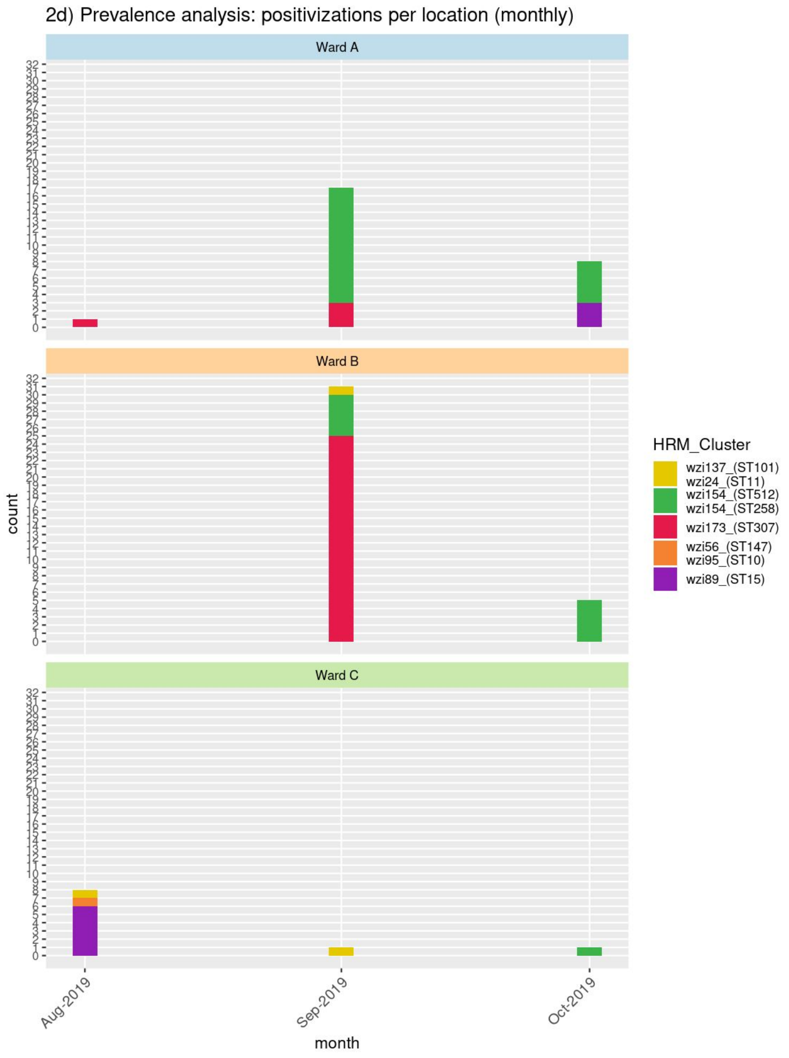

2e) Positivizations global (daily)

The bar charts show the location and the collection day for each positivization in all the locations (wards) combined. A positivization corresponds to the first time a patient is found positive to an isolate of a cluster. When two isolates of different clusters are isolated from a patient, that patient will have two positivization events (one per cluster). The position on the x axis corresponds to the positivization day.

High resolution file:
https://skynet.unimi.it/wp-content/uploads/MeltingPlot/files_supplementary_material/pdf_2e_big_positivization_global_daily.pdf

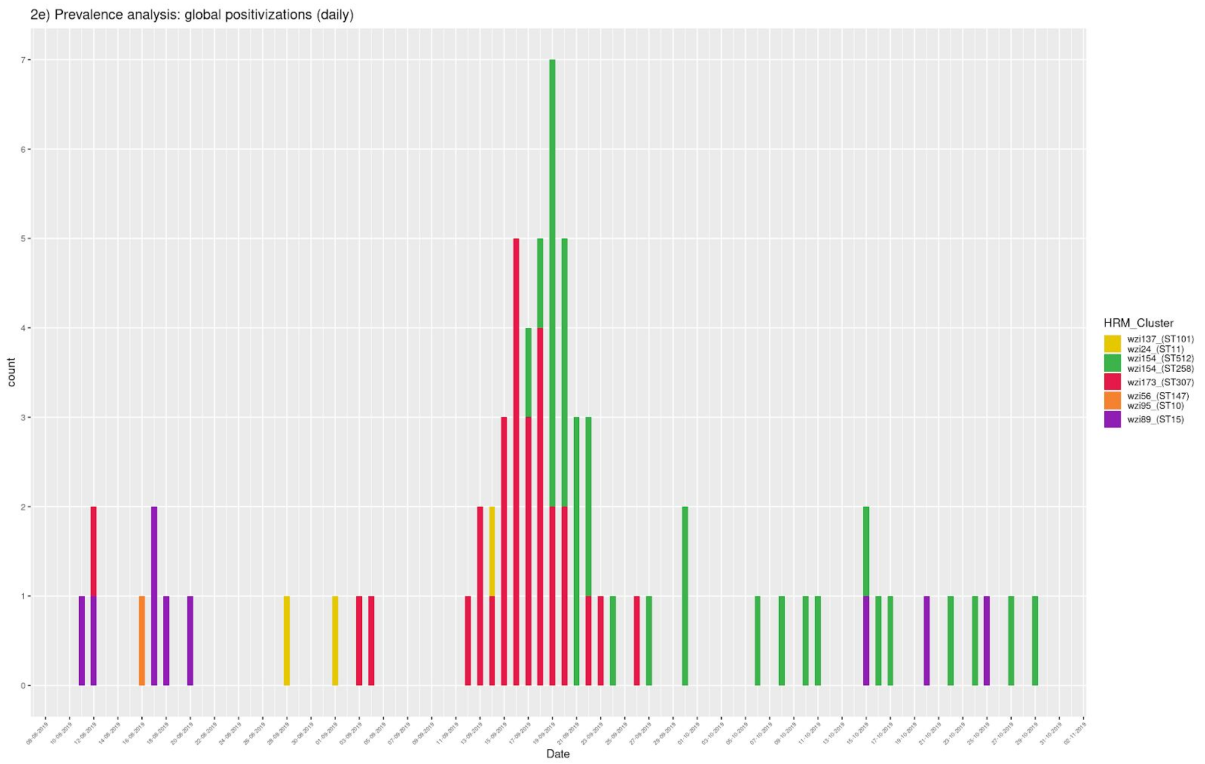

2f) Positivizations global (monthly)

The bar charts show the location and the collection month for each positivization in all the locations (wards) combined. This plot is created only if the collection date range is above 45 days. A positivization corresponds to the first time a patient is found positive to an isolate of a cluster. When two isolates of different clusters are isolated from a patient, that patient will have two positivization events (one per cluster). The position on the x axis corresponds to the positivization month.

High resolution file:
https://skynet.unimi.it/wp-content/uploads/MeltingPlot/files_supplementary_material/pdf_2f_big_positivization_global_monthly.pdf

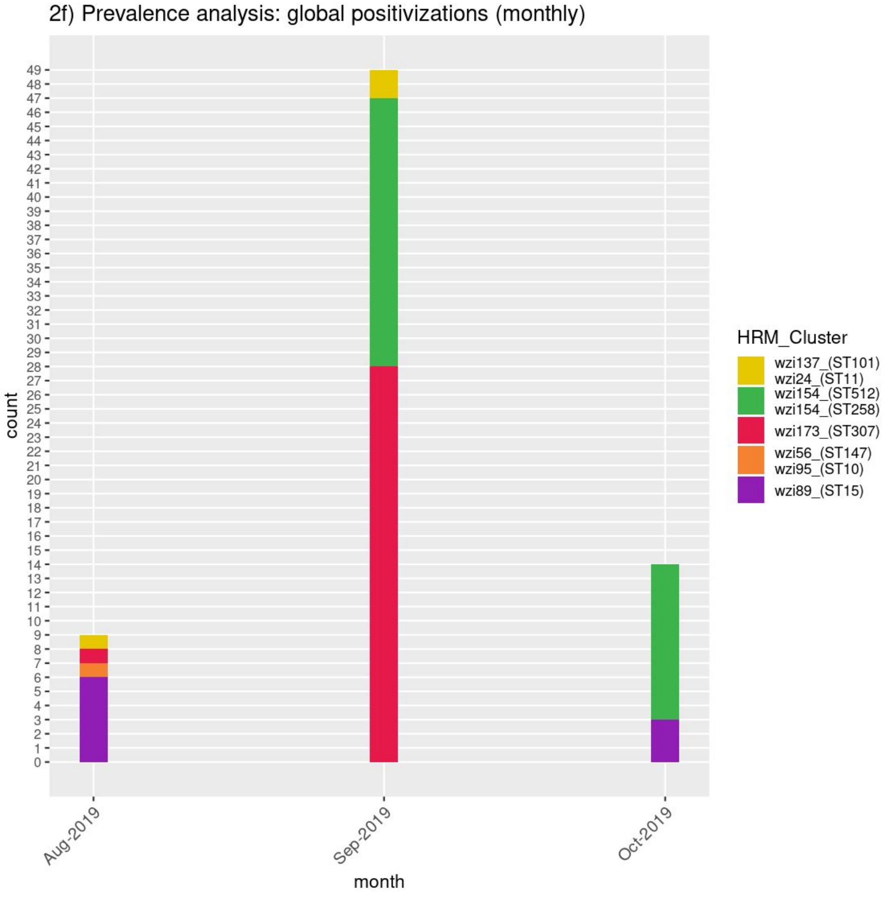

Transmission analysis

3a) Patients timeline

In this patients' timeline, each row refers to a patient and symbols represent the isolates. The shape of the symbol corresponds to the patient location (ward) at the time of isolate collection, the color represents the isolate cluster. Larger symbols represent positivizations: the first time a patient is found positive to an isolate of a cluster. If two or more isolates of the same cluster are isolated from a patient, that patient will have a first larger symbol (representing the positivization) and other smaller symbols (the other isolations). When two isolates of different clusters are isolated from the same patient, that patient will have two positivization and thus two larger symbols, one per cluster. In the plot, patients are sorted on the basis of the first isolation date.

High resolution file:
https://skynet.unimi.it/wp-content/uploads/MeltingPlot/files_supplementary_material/pdf_3a_big_patients_timeline.pdf

3a) Transmission analysis: patients' timeline

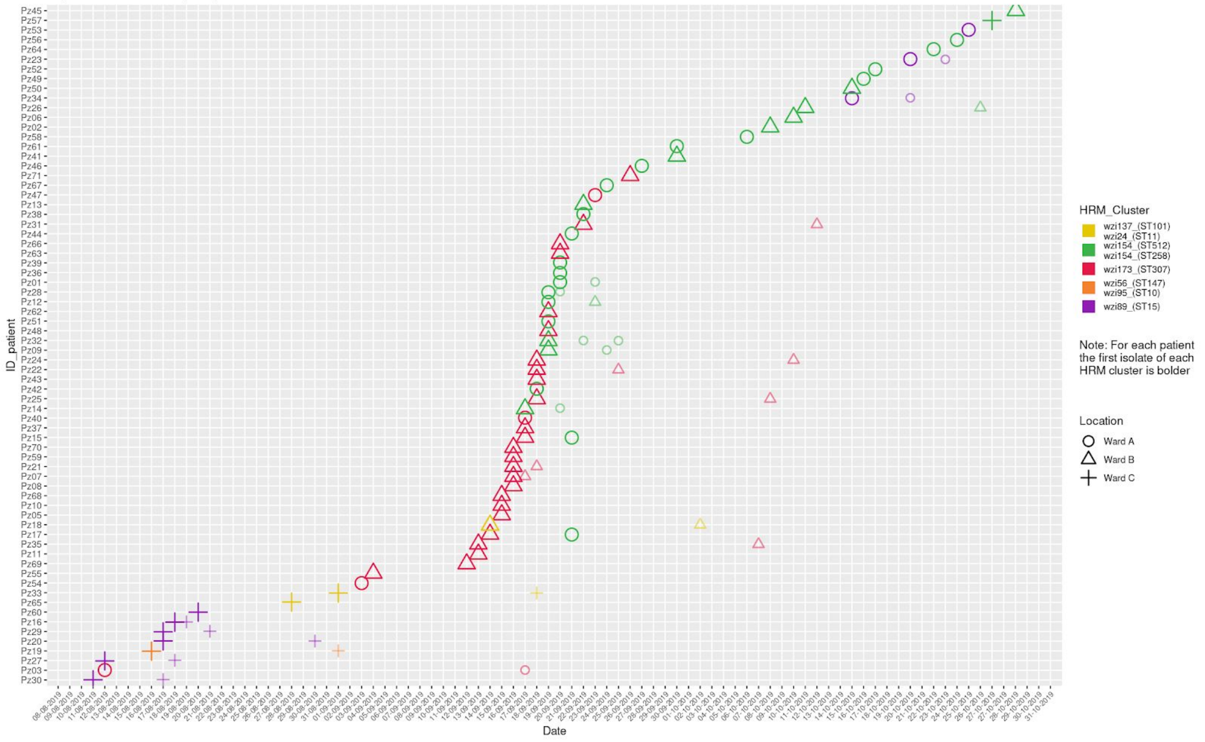

3b) Patient-to-patient graph

In this graph each vertex represent a patient and two vertices are connected if isolates of the same cluster are isolated from the two patients. Each patient is represented by a pie chart to show the visited locations (wards). The edges are thicker when the isolates of the same cluster were collected in the same location (ward) in less than 7 days (this amount of days can be set by the user, the default is 7).

High resolution file:
https://skynet.unimi.it/wp-content/uploads/MeltingPlot/files_supplementary_material/pdf_3b_big_patients_graph.pdf

3b) Transmission analysis: patient-to-patient graph

Note: links of connections occurrend within 7 days are bolder

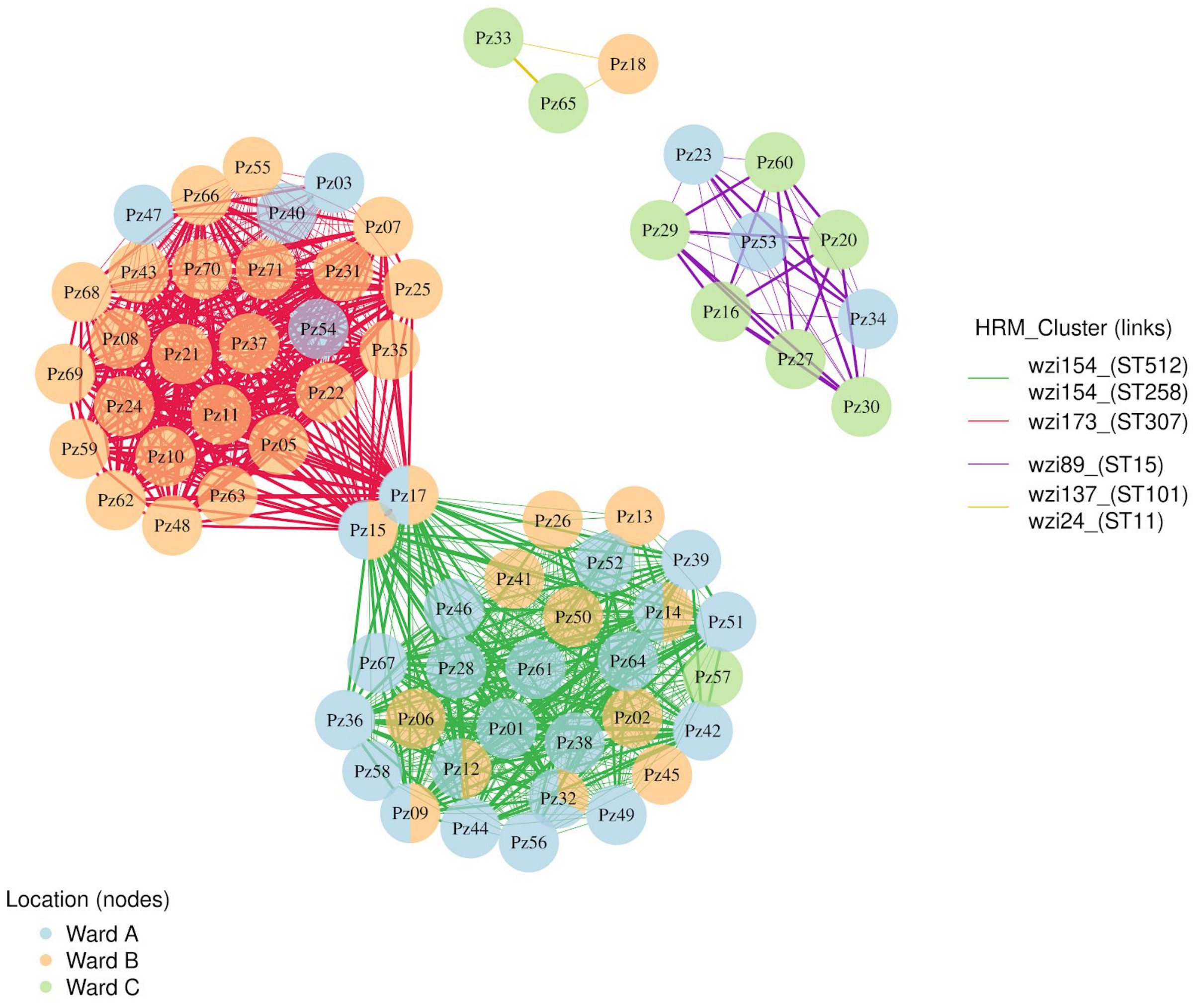

Tabular outputs

Isolates cluster table

This xls spreadsheet lists all the isolates, their HRM cluster, the betweenness values and cluster colors in hexadecimal code. Data and colors are those used to build the isolate graph produced in the HRM-based clustering/typing analysis.

Complete xls file:
https://skynet.unimi.it/wp-content/uploads/MeltingPlot/files_supplementary_material/table_big_clusters.xls

	A	B	C	D
1	ID_isolate	HRM_Cluster	Betweenness	color_hexa
2	49BG	wzi56_(ST147) & wzi95_(ST10)	0	#f58231
3	52BG	wzi56_(ST147) & wzi95_(ST10)	0	#f58231
4	BG-Kpn-25-18	wzi154_(ST512) & wzi154_(ST258)	0	#3cb44b
5	Is01	wzi154_(ST512) & wzi154_(ST258)	0	#3cb44b
6	Is02	wzi154_(ST512) & wzi154_(ST258)	0	#3cb44b
7	Is03	wzi154_(ST512) & wzi154_(ST258)	0	#3cb44b
8	Is04	wzi173_(ST307)	0.000322765947766	#e6194B
9	Is05	wzi89_(ST15)	0	#911eb4
10	Is06	wzi173_(ST307)	0.000322765947766	#e6194B
11	Is07	wzi173_(ST307)	0.000322765947766	#e6194B
12	Is08	wzi154_(ST512) & wzi154_(ST258)	0	#3cb44b
13	Is09	wzi173_(ST307)	0.000322765947766	#e6194B
14	Is10	wzi173_(ST307)	0.000322765947766	#e6194B
15	Is11	wzi173_(ST307)	0.000322765947766	#e6194B
16	Is12	wzi154_(ST512) & wzi154_(ST258)	0	#3cb44b
17	Is13	wzi154_(ST512) & wzi154_(ST258)	0	#3cb44b
18	Is14	wzi154_(ST512) & wzi154_(ST258)	0	#3cb44b
19	Is15	wzi173_(ST307)	0.000322765947766	#e6194B
20	Is16	wzi173_(ST307)	0.000322765947766	#e6194B
21	Is17	wzi154_(ST512) & wzi154_(ST258)	0	#3cb44b
22	Is19	wzi154_(ST512) & wzi154_(ST258)	0	#3cb44b
23	Is20	wzi173_(ST307)	0.000322765947766	#e6194B
24	Is21	wzi89_(ST15)	0	#911eb4
25	Is22	wzi173_(ST307)	0.000322765947766	#e6194B

Isolates cluster and patients table

This xls spreadsheet lists all the isolates and their relations to patients. Patient's ID, isolation data, location (ward), HRM cluster, betweenness values and cluster color (in hexadecimal code) are reported for every isolate. This information is used in the Prevalence analysis and the Transmission analysis.

Complete xls file:
https://skynet.unimi.it/wp-content/uploads/MeltingPlot/files_supplementary_material/table_big_clusters_patients.xls

	A	B	C	D	E	F	G
1	ID_isolate	ID_patient	Date	Location	HRM_Cluster	Betweenness	color_hexa
2	Is01	Pz01	9/20/2019	Ward A	wzi154_(ST512) & wzi154_(ST258)	0	#3cb44b
3	Is02	Pz01	9/23/2019	Ward A	wzi154_(ST512) & wzi154_(ST258)	0	#3cb44b
4	Is03	Pz02	10/8/2019	Ward B	wzi154_(ST512) & wzi154_(ST258)	0	#3cb44b
5	Is04	Pz03	8/12/2019	Ward A	wzi173_(ST307)	0.000322766	#e6194B
6	Is05	Pz34	10/15/2019	Ward A	wzi89_(ST15)	0	#911eb4
7	Is06	Pz03	9/17/2019	Ward A	wzi173_(ST307)	0.000322766	#e6194B
8	Is07	Pz05	9/15/2019	Ward B	wzi173_(ST307)	0.000322766	#e6194B
9	Is08	Pz06	10/10/2019	Ward B	wzi154_(ST512) & wzi154_(ST258)	0	#3cb44b
10	Is09	Pz07	9/17/2019	Ward B	wzi173_(ST307)	0.000322766	#e6194B
11	Is10	Pz08	9/16/2019	Ward B	wzi173_(ST307)	0.000322766	#e6194B
12	Is11	Pz07	9/16/2019	Ward B	wzi173_(ST307)	0.000322766	#e6194B
13	Is12	Pz09	9/24/2019	Ward A	wzi154_(ST512) & wzi154_(ST258)	0	#3cb44b
14	Is13	Pz09	9/19/2019	Ward B	wzi154_(ST512) & wzi154_(ST258)	0	#3cb44b
15	Is14	Pz32	9/25/2019	Ward A	wzi154_(ST512) & wzi154_(ST258)	0	#3cb44b
16	Is15	Pz10	9/15/2019	Ward B	wzi173_(ST307)	0.000322766	#e6194B
17	Is16	Pz11	9/13/2019	Ward B	wzi173_(ST307)	0.000322766	#e6194B
18	Is17	Pz12	9/23/2019	Ward B	wzi154_(ST512) & wzi154_(ST258)	0	#3cb44b
19	Is19	Pz14	9/20/2019	Ward A	wzi154_(ST512) & wzi154_(ST258)	0	#3cb44b
20	Is20	Pz15	9/17/2019	Ward B	wzi173_(ST307)	0.000322766	#e6194B
21	Is21	Pz16	8/18/2019	Ward C	wzi89_(ST15)	0	#911eb4
22	Is22	Pz17	9/14/2019	Ward B	wzi173_(ST307)	0.000322766	#e6194B
23	Is23	Pz18	9/14/2019	Ward B	wzi137_(ST101) & wzi24_(ST11)	0	#e6c800
24	Is24	Pz19	9/1/2019	Ward C	wzi56_(ST147) & wzi95_(ST10)	0	#f58231

References

[1] M. Perini *et al.*, “EasyPrimer: user-friendly tool for pan-PCR/HRM primers design. Development of an HRM protocol on *wzi* gene for fast *Klebsiella pneumoniae* typing,” *Sci. Rep.*, vol. 10, no. 1, p. 1307, Jan. 2020, doi: 10.1038/s41598-020-57742-z.

[2] M. E. J. Newman and M. Girvan, “Finding and evaluating community structure in networks,” *Physical Review E*, vol. 69, no. 2. 2004, doi: 10.1103/physreve.69.026113.

[3] G. Csardi, T. Nepusz, and Others, “The igraph software package for complex network research,” *InterJournal, complex systems*, vol. 1695, no. 5, pp. 1–9, 2006, [Online]. Available: http://igraph.org.
